# Assessing the Subjective Happiness of Parents of Children With Severe Motor and Intellectual Disabilities Receiving Home Care

**DOI:** 10.7759/cureus.64562

**Published:** 2024-07-15

**Authors:** Yuto Arai, Ryuki Kadekaru, Tohru Okanishi, Akiko Tamasaki, Yoshihiro Maegaki

**Affiliations:** 1 Child Neurology, Tottori University Hospital, Yonago, JPN; 2 Psychology, Advanced Medicine, Innovation and Clinical Research Center, Tottori University Hospital, Yonago, JPN; 3 Child Neurology, Home Care Support Clinic, Yonago, JPN; 4 Child Neurology, Institute of Neurological Sciences, Faculty of Medicine, Tottori University, Tottori, JPN

**Keywords:** motor disabilities, permanent tracheostomy, subjective happiness, intellectual disability (id), psychological care, parental sex

## Abstract

Background: Home care for children with severe motor and intellectual disabilities (SMID) is challenging for parents because it is highly intensive and long-lasting. The pursuit of happiness is an essential goal for everyone. However, only a few studies have focused on the happiness of families with such children.

Objective: The study aimed to examine the subjective happiness of parents of children with SMID receiving home care and identify the factors associated with their happiness.

Methods: We conducted a cross-sectional online questionnaire-based survey of 23 parents of children with SMID and nurses with children without disabilities as controls at Tottori University Hospital, Yonago, Japan from July 1 to August 31, 2023. We set the subjective happiness scale (SHS) scores as the outcomes. We used the Mann-Whitney U test to compare the SHS scores between the two groups. Moreover, we extracted the clinical and demographic factors affecting the SHS scores of parents of children with SMID using univariate linear regression analysis.

Results: We obtained responses from 12 parents with SMID and 105 controls. The average SHS scores of parents with SMID and controls were 4.8 and 4.7, respectively, and both groups did not differ significantly. Univariate analysis showed that parental male sex and the presence of a tracheostomy were negatively associated with the SHS scores of parents.

Conclusions: The SHS scores did not differ significantly between parents with SMID and controls. However, more attention seemed necessary for fathers and parents of children who have undergone tracheostomies. Given the exploratory nature of this study and its small sample size, larger-scale investigations are warranted. Additionally, qualitative research conducted after establishing trustful relationships could provide further insights.

## Introduction

Happiness is a subjective emotion measured by the degree to which a person feels satisfied with their life [1,2]. A person's sense of happiness has widespread implications for their ability to function in all aspects of life. Happier people respond better to crises and do not dwell on negative emotions or self-blame. They are more flexible and do not give up on their set goals [3].

In general, parents of children with disabilities tend to report higher rates of stress, anxiety, and depression compared to the average population [4,5]. However, several studies have documented that raising a child with special needs can indeed positively impact the family framework [6] and enhance parental relationships by fostering greater cooperation [7]. This sense of teamwork certainly strengthens spousal bonds [6,7]. Therefore, it cannot be definitively asserted that the mental well-being of parents raising children with disabilities is universally low.

Severe motor and intellectual disabilities (SMID) are a heterogeneous group of disorders that include profound physical or mental disabilities [8]. Children with such disorders require permanent human and technical assistance throughout their lives due to various severe disabilities and multiple comorbidities [9]. Many children suffer from physical difficulties with food ingestion, which necessitates gastrointestinal tube feeding and causes gastroesophageal reflux, leading to chronic respiratory disorders and recurrent pneumonia [10]. Raising these children at home may prove challenging for parents because the caring task is highly intensive and long-lasting [11]. Caregivers have reported deteriorating health status, financial difficulties, restrictions in social participation, and physical overload [9,11-13]. The pursuit of happiness is an essential goal for everyone [14]; however, only a few studies have focused on happiness among families of children with SMID. Understanding the happiness of parents of children with SMID enables the provision of support and effective policy programs to enhance their happiness. For instance, in France, a study investigated the quality of life (QoL) of healthcare professionals involved in the care of children with SMID; the study identified income and age as factors influencing QoL and suggested improvements in workplace settings [15].

This study aimed to examine the happiness of home-caring parents of children with SMID. The primary outcome was the difference in the subjective happiness scale (SHS) scores in these parents compared to the scores of parents of children without SMID. The secondary outcomes were the clinical factors that influenced the SHS scores among parents of children with SMID.

## Materials and methods

Participants and procedures

As of 2021, there were 139 individuals with SMID in the Tottori Prefecture [16]. The Department of Pediatric Neurology at the Tottori University School of Medicine's affiliated hospital provides support for home-based medical care [17]. We introduced our study to parents of children with SMID with a history of outpatient visits to our hospital by handing out flyers about this research. In order to assess the happiness of home-caring parents of children with SMID, we recruited nurses who had children without disabilities at our hospital as controls using posters placed at the hospital. We conducted a cross-sectional online questionnaire-based survey from July 1, 2023, to August 31, 2023. The survey was available online, and parents of children with SMID and the recruited nurses accessed and answered it through a QR code. Participation in this study was voluntary, and information was collected anonymously after obtaining consent from each respondent to ensure confidentiality throughout data collection. This study was approved by the Institutional Ethics Committee of Tottori University Hospital (approval number: 23A025) on May 19, 2023.

SHS

The SHS is designed to measure an individual's subjective sense of happiness [18]. This scale comprises four questions that prompt participants to assess the extent to which they perceive themselves as happy compared to others. The SHS consists of the following four items, which are rated on a 7-point Likert-type scale: Item 1: ‘‘In general, I consider myself: (1) not a very happy person, (7) a very happy person’’; Item 2: ‘‘Compared to most of my peers, I consider myself: (1) less happy, (7) more happy’’; Item 3: ‘‘Some people are generally very happy. They enjoy life regardless of what is going on, getting the most out of everything. To what extent does this characterization describe you? (1) not at all, (7) a great deal’’; and Item 4: ‘‘Some people are generally not very happy. Although they are not depressed, they never seem as happy as they might be. To what extent does this characterization describe you? (1) not at all, (7) a great deal.’’ Item 4 is “reverse coded” with a descending sequence. Each participant's score is calculated by averaging their responses, with higher scores indicating a greater level of happiness. Recently, the SHS has been translated into Japanese, and it showed high internal consistency (Cronbach's α = 0.82) and test-retest reliability over five months (intraclass correlation coefficient = 0.86) [19]. We obtained permission to use the Japanese version of the SHS from its creators, Shimai et al. [19].

Data collection

We collected information through a questionnaire, including participants' gender, age, and annual income. We inquired about the frequency of airway suctioning, enemas, and repositioning and the time taken for a single meal in parents with children with SMID. In addition, we collected data on the number of family members assisting in care besides the respondent, the burden of care (ranging from 1 for "not at all burdensome" to 5 for "extremely burdensome"), and satisfaction with social resources and economic situation (ranging from 1 for "not satisfied at all" to 5 for "extremely satisfied"). Furthermore, we obtained information from medical records regarding the Oshima classification, the presence of a tracheostomy, and the method of enteral nutrition.

Statistical analysis

We compared the data between parents with children with disabilities and controls. We compared categorical variables using Fisher's exact test. In contrast, ordinal or continuous variables were compared using the Mann-Whitney U test. We assessed the association between the clinical factors and SHS scores of parents using univariate linear regression analysis. Statistical significance for all analyses was set at p < 0.05, and instances of 0.05 ≤ p < 0.10 were considered as trends. Multivariate analysis was not performed due to sample size limitations and the study’s exploratory nature. Data analyses were performed using Statistical Package for the Social Sciences (IBM SPSS Statistics for Windows, IBM Corp., Version 25.0, Armonk, NY).

## Results

We obtained responses from 12 parents with children with disabilities (response rate: 52.2%) and 105 controls. Table 1 shows the participants’ characteristics. In the parents of children with SMID, the age distribution was as follows: two (16.7%) were aged 30-39 years; five (41.7%), 40-49 years; two (16.7%), 50-59 years; and three (25.0%), 60-69 years. Ten (83.3%) parents were women. Annual income was as follows: four (33.3%) were <4,000,000 yen/year, three (25.0%) were 4,000,000 to 5,999,000 yen/year, and one (8.3%) were ≥6,000,000 yen/year. In the parents of children without disabilities, the age distribution was as follows: 38 (36.2%) were aged 30-39 years; 42 (40.0%), 40-49 years; 22 (21.0%), 50-59 years; and three (2.8%), 60-69 years. A total of 98 (93.3%) parents were women. Annual income was as follows: 17 (16.2%) were <4,000,000 yen/year, 54 (51.5%) were 4,000,000 to 5,999,000 yen/year, and 18 (17.1%) were ≥6,000,000 yen/year. When age and annual income were treated as a continuous variable with four and three levels respectively, the Mann-Whitney U test showed no significant difference between the two groups (p = 0.063 and p = 0.10, respectively). In Fisher's exact test, there was no significant difference in gender between the two groups (p = 0.23).

**Table 1:** Parental background characteristics SMID: severe motor and intellectual disability

Background of parents		Parents of children with SMID		Controls		p-value
		n = 12		n = 105		
Age (years)	30-39	2 (16.7%)		38 (36.2%)		0.063
	40-49	5 (41.7%)		42 (40.0%)		
	50-59	2 (16.7%)		22 (21.0%)		
	60-69	3 (25.0%)		3 (2.8%)		
Sex	Male	2 (16.7%)		7 (6.7%)		0.23
	Female	10 (83.3%)		98 (93.3%)		
Annual income (10^3^ yen/year)	<4000	4 (33.3%)		17 (16.2%)		0.1
	4000 to 5999	3 (25.0%)		54 (51.5%)		
	≥6000	1 (8.3%)		18 (17.1%)		
	No answer	4 (33.3%)		16 (15.2%)		

Table 2 shows the characteristics of the children with disabilities. The average age was 17.9 (±11.9) years. We categorized 11 (91.7%) patients as class 1 as per the Oshima classification. Tracheostomy was performed in seven (58.3%) patients and a gastrostomy tube was placed in 10 (83.3%) patients. Notably, three (25%) patients required airway suctioning ≥10 times daily, whereas seven (58.3%) required repositioning at least thrice a day.

**Table 2:** Clinical characteristics of children with SMID SMID: severe motor and intellectual disability; SD: standard deviation

Characteristics of children with SMID (n = 12)		n (%)
		Mean (±SD)
Age (years)		17.9 (±11.9)
Sex	Male	5 (41.7%)
Severity (Oshima classification)	1	11 (91.7%)
	2	1 (8.3%)
	3	0 (0%)
	4	0 (0%)
Tracheostomy	Yes	7 (58.3%)
Enteral nutrition	Yes	12 (100%)
Gastrostomy	Yes	10 (83.3%)
Frequency of airway suction care	Unnecessary	3 (25.0%)
	1-4 times per day	4 (33.3%)
	5-9 times per day	2 (16.7%)
	>9 times per day	3 (25.0%)
Frequency of enema care	Unnecessary	2 (16.7%)
	1 time per day	8 (66.7%)
	2 times per day	1 (8.3%)
	>2 times per day	1 (8.3%)
Time required for one meal	<30 minutes	6 (50.0%)
	30-120 minutes	5 (41.7%)
	121-300 minutes	1 (8.3%)
Frequency of repositioning care	Unnecessary	1 (8.3%)
	1-2 times per day	4 (33.3%)
	>2 times per day	7 (58.3%)
Number of family members assisting in care besides the respondent	0	1 (9.1%)
	1	5 (45.5%)
	2	3 (27.2%)
	>2	2 (18.2%)
	No answer	1 (9.1%)
Burden of care		2.8 (±1.2)
Satisfaction with social resources		4.1 (±0.9)
Satisfaction with economic situation		2.5 (±0.85)

Comparison of SHS scores

**Figure 1:**
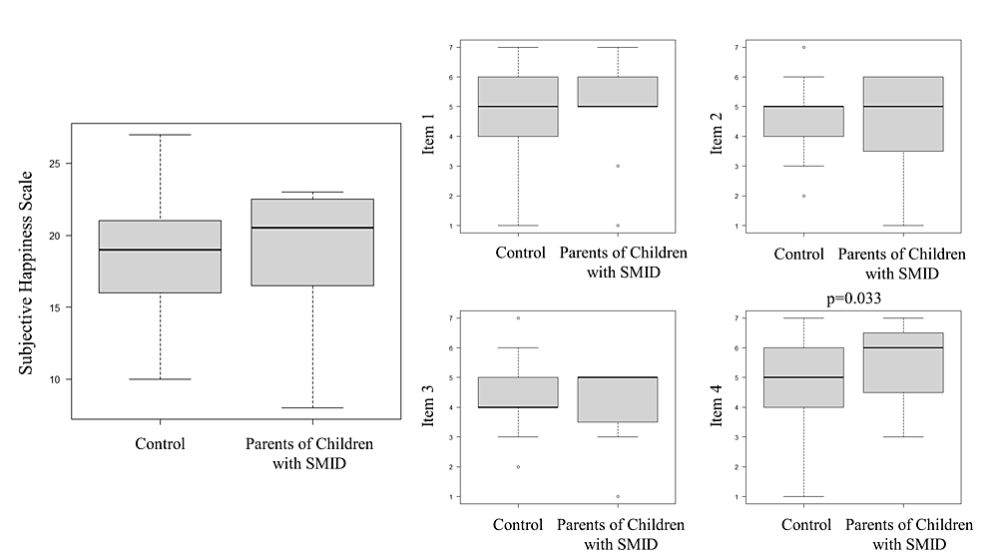
Comparison of subjective happiness scale scores SMID: severe motor and intellectual disabilities

The overall Cronbach's α reliability coefficient was 0.75 in our study. The average total SHS score of parents with children with SMID was 4.8 (±1.1), whereas that in the controls was 4.7 (±0.9), with no significant difference between the two groups (p = 0.45) (Figure 1). The average scores for items 1, 2, and 3 of the SHS were 4.8 (±1.6), 4.3 (±1.8), and 3.9 (±1.6) for parents of children with SMID and 4.9 (±1.2), 4.7 (±1.1), and 4.3 (±1.2) for the control group, with no significant difference between the two groups (p = 0.38, 0.92 and 0.74, respectively). However, the average score for item 4 of the SHS score was 5.5 (±1.3) for parents of children with SMID and 4.7 (±1.3) for the control group, with a significant difference between the two groups (p = 0.033).

Factors affecting the SHS scores of parents of children with SMID

Regarding parental characteristics (Table 3), univariate regression analysis showed a negative association between parental male sex and the SHS scores (regression coefficient (B), -1.8; 95% confidence interval (CI), -3.4, -0.19; p = 0.032). Moreover, the annual income was positively associated with the SHS scores (B, 0.71; 95% CI, -0.017, 1.4; p = 0.054). Regarding children’s characteristics, univariate regression analysis showed that the presence of a tracheostomy was negatively associated with the SHS scores (B, -1.4; 95% CI, -2.6, -0.16; p = 0.030). In addition, there was a trend showing that age was negatively associated with the SHS scores (B, -0.055; 95% CI, -0.11, 0.00081; p = 0.053).

**Table 3:** Univariate simple linear regression analysis to identify clinical factors associated with the SHS scores of parents of children with SMID SHS: subjective happiness scale; SMID: severe motor and intellectual disability; B: regression coefficient; CI: confidence interval; * p < 0.1; ** p < 0.05

Clinical factors	B	95% CI		p-value
Characteristics of parents				
Age	-0.37	-1.1	0.32	0.26
Sex (0=female, 1=male)	-1.8	-3.4	-0.19	0.032**
Annual income	0.71	-0.017	1.4	0.054*
Number of family members assisting in care besides the respondent	0.18	-0.73	1.1	0.67
Burden of care	0.1	-0.56	0.75	0.76
Satisfaction with social resources	-0.34	-1.2	0.52	0.4
Satisfaction with economic situation	0.38	-0.68	1.5	0.43
Characteristics of children				
Age	-0.055	-0.11	0.0008	0.053*
Sex (0=female, 1=male)	-0.69	-2.2	0.79	0.32
Tracheostomy (0=no, 1=yes)	-1.4	-2.6	-0.16	0.030**
Gastrostomy (0=no, 1=yes)	-1.2	-3.1	0.67	0.18
Frequency of airway suction care	-0.39	-1.02	0.24	0.2
Frequency of enema care	-0.036	-1.04	1	0.94
Time required for one meal	-0.76	-1.8	0.3	0.14
Frequency of repositioning care	-0.35	-1.5	0.81	0.52

## Discussion

We conducted an exploratory study on the subjective happiness of parents with children with SMID. The results showed no significant difference in happiness between such parents and controls. However, the parental male sex and the presence of a tracheostomy in the child were negatively associated with the SHS scores. In addition, there was a trend showing that parental income had a positive correlation, whereas the child's age correlated negatively with the SHS scores of such parents.

The happiness levels of these parents did not differ significantly from those of the control group. Our results did not align with previous reports showing low psychological QoL among parents of children with severe disabilities [9,13]. In contrast, parents of children with disabilities, while experiencing suffering and sorrow, also reported feelings of strength, joy, hope, and love in previous studies [20]. Notably, some reports have indicated that parents gain a sense of accomplishment, become better individuals, and feel that they have done their best for their children [21]. In addition, some reports assert that children with disabilities often serve as sources of happiness [22,23]. Findler et al. insist that it is essential for parents of children with disabilities to pursue happiness and for society to provide support in this pursuit [2]. Therefore, society and healthcare professionals should continually support the needs of such parents.

Fathers of children with disabilities had lower levels of happiness than mothers. Davis et al. conducted a psychological study on parents of children with cerebral palsy. They found that mothers often experienced guilt and may lean towards overprotectiveness, becoming deeply engrossed in an intense relationship with the child. In contrast, fathers tend to adopt a broader perspective, focusing on the well-being of the entire family, paying attention to the needs of their other children, and working towards reestablishing a sense of normalcy in the family's functioning [24]. Mothers often present with their children during routine outpatient visits, allowing healthcare professionals to understand their needs better. However, fathers who are unable to attend these visits because of work or other reasons may also bear the pressure of supporting the family, and healthcare professionals may need to consider the concerns of such fathers about psychological well-being, especially regarding their family functioning.

Lower QoL among parents of children who have undergone tracheostomy has been reported [25-27], and our study supports this finding. Tracheostomies are artificial airways for patients whose natural airway patency or safety is compromised or for those who require prolonged support with ventilation or secretion clearance [25,26]. However, parents face significant challenges and are daunted by the responsibilities associated with caring for a child with a tracheostomy [25,26]. Adapting to these responsibilities places a substantial burden on caregivers, affecting their lifestyle and family dynamics [26]. Therefore, clinicians should consider the substantial psychosocial impact on parents [27]. Constant psychosocial support may be required, particularly for families with children who have undergone tracheostomies.

This study had some limitations. First, due to the small sample size, our study has insufficient replicability and did not adequately address confounding factors. However, to the best of our knowledge, this is the first study to compare the SHS scores of parents of children with SMID to those of a general population and to identify factors associated with the SHS scores, making it a valuable contribution to the literature. Building on this exploratory study, it is hoped that larger-scale investigations will be conducted in the future. Second, the data for this study were derived from self-report scales, which are affected by the method’s bias. For instance, there is a possibility that parents of children with SMID may have scored higher on the SHS due to social desirability bias [28], aiming to present themselves in a more favorable light to others. Therefore, to mitigate this bias, it is considered useful to establish a trustful relationship between researchers and participants to encourage honest responses. Similarly, a qualitative research approach may be considered for future study. Third, the validity of the control group may limit the generalization of findings. We selected nurses with children without disabilities as our control group. Happiness among healthcare professionals is influenced by both personal and organizational factors [29]. In a large-scale study examining the SHS score of over 2,000 Japanese adults aged ≥20 years, the average SHS score across all ages was 4.2 [30]. Therefore, not only did parents with children with SMID show higher SHS values, but the control group also demonstrated SHS values higher than that of the average Japanese population. While caution is warranted regarding the validity of the control group, this finding suggests that the happiness of parents of children with SMID may not necessarily be lower.

## Conclusions

We conducted an exploratory study on the subjective happiness of parents of children with SMID. The SHS scores in such parents did not differ significantly from those in controls. Nevertheless, it is crucial for society and healthcare professionals to support parents of children with SMID in pursuing happiness, especially fathers of children with SMID and parents of children who have undergone tracheostomies. It would be valuable to conduct a large-scale survey study with the general population as a control group, building upon the findings of this study to investigate the happiness levels of parents of children with SMID. Additionally, qualitative research conducted after establishing trustful relationships could provide further insights.

## References

[1] Diener E (2000). Subjective well-being. The science of happiness and a proposal for a national index. Am Psychol.

[2] Findler L, Klein Jacoby A, Gabis L (2016). Subjective happiness among mothers of children with disabilities: the role of stress, attachment, guilt and social support. Res Dev Disabil.

[3] Abbe A, Tkach C, Lyubomirsky S (2003). The art of living by dispositionally happy people. J Happiness Stud.

[4] Robinson S, Hastings RP, Weiss JA, Pagavathsing J, Lunsky Y (2018). Self-compassion and psychological distress in parents of young people and adults with intellectual and developmental disabilities. J Appl Res Intellect Disabil.

[5] Heifetz M, Brown HK, Chacra MA, Tint A, Vigod S, Bluestein D, Lunsky Y (2019). Mental health challenges and resilience among mothers with intellectual and developmental disabilities. Disabil Health J.

[6] Green SE (2007). "We're tired, not sad": benefits and burdens of mothering a child with a disability. Soc Sci Med.

[7] Olsson MB, Hwang CP (2001). Depression in mothers and fathers of children with intellectual disability. J Intellect Disabil Res.

[8] Kurihara M, Kumagai K, Noda Y, Watanabe M, Imai M (1998). Prognosis in severe motor and intellectual disabilities syndrome complicated by epilepsy. Brain Dev.

[9] Rousseau MC, Baumstarck K, Valkov M (2020). Impact of severe polyhandicap cared for at home on French informal caregivers' burden: a cross-sectional study. BMJ Open.

[10] Zijlstra HP, Vlaskamp C (2005). The impact of medical conditions on the support of children with profound intellectual and multiple disabilities. J Appl Res Intellect Disabil.

[11] Tadema AC, Vlaskamp C (2010). The time and effort in taking care for children with profound intellectual and multiple disabilities: a study on care load and support. Br J Learn Disabil.

[12] Chou YC, Chiao C, Fu LY (2011). Health status, social support, and quality of life among family carers of adults with profound intellectual and multiple disabilities (PIMD) in Taiwan. J Intellect Dev Disabil.

[13] Rousseau MC, Baumstarck K, Khaldi-Cherif S (2019). Impact of severe polyhandicap on parents' quality of life: a large French cross-sectional study. PLoS One.

[14] Lyubomirsky S, Sheldon KM, Schkade D (2005). Pursuing happiness: the architecture of sustainable change. Rev Gen Psychol.

[15] Rousseau MC, Baumstarck K, Leroy T (2017). Impact of caring for patients with severe and complex disabilities on health care workers' quality of life: determinants and specificities. Dev Med Child Neurol.

[16] (2023). Tottori prefecture. https://www.pref.tottori.lg.jp/299144.htm.

[17] (2023). Division of Child Neurology, Institute of Neurological Science, Faculty of Medicine, & Tottori University. https://www.med.tottori-u.ac.jp/nousho/index.php.

[18] Lyubomirsky S, Lepper HS (1999). A measure of subjective happiness: preliminary reliability and construct validation. Soc Indic Res.

[19] Shimai S, Otake K, Utsuki N, Ikemi A, Lyubomirsky S (2004). Development of a Japanese version of the Subjective Happiness Scale (SHS), and examination of its validity and reliability [Article in Japanese]. Nihon Koshu Eisei Zasshi.

[20] Kearney PM, Griffin T (2001). Between joy and sorrow: being a parent of a child with developmental disability. J Adv Nurs.

[21] Hastings RP, Taunt HM (2002). Positive perceptions in families of children with developmental disabilities. Am J Ment Retard.

[22] Greer FA, Grey IM, McClean B (2006). Coping and positive perceptions in Irish mothers of children with intellectual disabilities. J Intellect Disabil.

[23] Hastings RP, Allen R, McDermott K, Still D (2002). Factors related to positive perceptions in mothers of children with intellectual disabilities. J Appl Res Intellect Disabil.

[24] Davis E, Shelly A, Waters E, Boyd R, Cook K, Davern M, Reddihough D (2010). The impact of caring for a child with cerebral palsy: quality of life for mothers and fathers. Child Care Health Dev.

[25] Westwood EL, Hutchins JV, Thevasagayam R (2019). Quality of life in paediatric tracheostomy patients and their caregivers - a cross-sectional study. Int J Pediatr Otorhinolaryngol.

[26] Hopkins C, Whetstone S, Foster T, Blaney S, Morrison G (2009). The impact of paediatric tracheostomy on both patient and parent. Int J Pediatr Otorhinolaryngol.

[27] Chandran A, Sikka K, Thakar A, Lodha R, Irugu DVK, Kumar R, Sharma SC (2021). The impact of pediatric tracheostomy on the quality of life of caregivers. Int J Pediatr Otorhinolaryngol.

[28] Meisters J, Hoffmann A, Musch J (2020). Controlling social desirability bias: an experimental investigation of the extended crosswise model. PLoS One.

[29] Brennan EJ (2017). Towards resilience and wellbeing in nurses. Br J Nurs.

[30] Shimai S, Yamamiya Y, Fukuda S (2018). Subjective happiness among Japanese adults: an upward tendency associated with age [Article in Japanese]. Nihon Koshu Eisei Zasshi.

